# Comparative Diagnostic Performance of TST and IGRAs in the Diagnosis of Latent Tuberculosis Infection: A Systematic Review and Diagnostic Meta-Analysis

**DOI:** 10.3390/diagnostics16060951

**Published:** 2026-03-23

**Authors:** Shyamkumar Sriram, Tareq Abualfaraj, Manal Ali Alsharif, Marwa Zalat, Saad Madani Alawfi, Hammad Ali Fadlalmola, Muayad Albadrani

**Affiliations:** 1 Department of Rehabilitation and Health Services, College of Health and Public Service, University of North Texas, Denton, TX 76203, USA; 2 Department of Basic Medical Sciences, College of Medicine, Taibah University, Madinah 42353, Saudi Arabia; 3 Health and Life Research Center, Taibah University, Madinah 42353, Saudi Arabia; 4 Training, National and International Collaboration, Academic Affairs, Madinah Health Cluster, Madinah 42319, Saudi Arabia; 5 Department of Family and Community Medicine and Medical Education, College of Medicine, Taibah University, Madinah 42353, Saudi Arabia; 6 Internal Medicine Department, Prince Sultan Armed Forces Hospital in Madinah, Madinah 42375, Saudi Arabia; 7 Department of Community Health Nursing, Nursing College, Taibah University, Madinah 42353, Saudi Arabia

**Keywords:** tuberculosis, latent tuberculosis infection, tuberculin skin test, interferon-gamma release assay, TST, IGRA

## Abstract

**Background:** Patients with latent tuberculosis infection are mainly asymptomatic, but they still carry a notable risk of developing active TB, particularly when the host becomes immunosuppressed. Hence, appropriate diagnosis and management for LTBI are essential. Tuberculin skin test (TST) and interferon-gamma release assays (IGRAs) are among the most commonly utilized methods for detecting LTBI. Until now, no agreement has been established regarding the most effective diagnostic test, either TST or IGRA, so our study aims to evaluate the diagnostic utility of TST versus IGRA in detecting LTBI. **Methods:** An extensive literature search was executed in several databases from inception till June 2024. We included all the available studies that compared TST versus IGRA concurrently applied to the same study participants, utilizing one of the following proxy reference standards: previous contact with a tuberculosis patient, tuberculosis history, chest x-ray suggestive of tuberculosis, or a combination of them. The sensitivity (SN) and specificity (SP) were imputed with their 95% confidence interval (CI). A bivariate random-effects model within the OpenMeta-Analyst software was utilized for data analysis. **Results:** We included 39 studies, and our primary analysis regarding LTBI revealed that TST has an SN of 0.320 (95% CI [0.254–0.393]) and an SP of 0.808 (95% CI [0.752–0.854]). Nevertheless, the IGRA exhibited a higher SN estimated at 0.362 (95% CI [0.295–0.434]) and a lower SP estimated at 0.758 (95% CI [0.700–0.808]). Regarding the adult population, TST consistently showed a lower SN and a higher SP relative to IGRA. However, within the pediatric population, TST showed higher SN and lower SP when compared to IGRA. Furthermore, TST also showed a lower SN and a higher SP within hemodialysis and organ transplant patients than IGRA. **Conclusions:** Our diagnostic test meta-analysis revealed that TST was associated with a lower SN and a higher SP than IGRA. Clinicians should interpret these findings with caution, considering the substantial heterogeneity observed across the included studies, the reliance on proxy reference standards, the potential influence of BCG vaccination status, and the considerable overlap in confidence intervals between TST and IGRA estimates across most analyses.

## 1. Introduction

Tuberculosis (TB), a significant health concern caused by mycobacterium tuberculosis, infects around 25% of the world population. According to the World Health Organization (WHO) Global Tuberculosis Report 2024, an estimated 10.8 million people developed TB disease and approximately 1.25 million deaths were attributed to TB worldwide in 2023, making it once again the leading cause of death from a single infectious agent globally, surpassing COVID-19 [1]. The WHO Global Tuberculosis Report 2025 further confirmed these trends, reporting 10.7 million incident cases in 2024 [2]. Over the past several decades, the sustained efforts and investments of governmental and non-governmental committees have resulted in a steady decline in this disease’s incidence and mortality rates, although the COVID-19 pandemic substantially disrupted these gains between 2020 and 2022 [1]. Over the past several decades, the sustained efforts and investments of governmental and non-governmental committees have resulted in a steady decline in this disease’s incidence and mortality rates [2].

Within immunocompetent individuals, primary tuberculosis infection can be controlled but not completely eradicated, enabling the organism to persist in a dormant state within the patient for several years. This condition of dormant tuberculosis infection, identified as latent tuberculosis infection (LTBI), is often defined by the asymptomatic existence of tuberculosis bacteria within the lung parenchyma without radiologic or clinical evidence of active tuberculosis [3]. Furthermore, ensuring an accurate latent tuberculosis diagnosis is underscored by the danger of its activation to an active and contagious disease, especially under conditions of host immunosuppression due to comorbid illness or medical therapy [4]. Hence, in addition to diagnosing active TB, the identification and treatment of LTBI are crucial elements for effective TB control [5].

The absence of a definitive diagnostic gold standard further challenges the accurate diagnosis of LTBI. Commonly used criteria involve previous contact with an infected person, active TB history, or chest x-ray findings suggestive of active disease [3]. While the tuberculin skin test (TST) continues to be the most frequently utilized test, its effectiveness is limited in Bacille Calmette-Guérin (BCG) vaccinated individuals, as cell-mediated immunity to tuberculin antigens can be indicative of prior exposure to Mycobacterium bovis bacilli found in the BCG vaccine. Furthermore, such cell-mediated immunity could reflect past exposure to similar antigens from environmental mycobacteria or a prior tuberculosis infection that was eradicated either through immune strategies or medical management [6]. To achieve better diagnostic specificity, interferon-gamma release assays (IGRAs), encompassing QuantiFERON Gold (QFT-G) and T. SPOT.TB) have emerged. These assays detect the interferon (IFN) gamma released by factor T cells in response to stimulation with Mycobacterium antigens, particularly early secreted antigenic target 6 and culture filtrate protein [7,8]. Given that these antigens are highly specific to M. tuberculosis and are absent in BCG strains, IGRAs are unaffected by BCG vaccination and may represent a superior alternative to TST for diagnosing LTBI [9].

Previous meta-analyses comparing TST and IGRAs revealed conflicting results regarding the sensitivity and specificity of both tests; for instance, Ferguson et al. [4] found that IGRAs had higher sensitivity, but TST showed lower specificity in hemodialysis patients, whereas Auguste et al. [10] reported that neither test was clearly superior, with wide and overlapping credible intervals across varied high-risk populations. These discrepancies may be attributed to differences in study populations, inclusion criteria, reference standards, and analytical methods employed [4,10]. Hence, we conducted this study to evaluate the diagnostic utility of TST and IGRAs for detecting LTBI.

## 2. Methods

Our research was executed in strict accordance with Cochrane Handbook rules [11]. PRISMA statement principles were followed during the reporting of this study [12].

### 2.1. Selection Criteria

Studies were involved in our diagnostic meta-analysis if they met all of our inclusion criteria. First, individuals with LTBI are determined by previous contact with tuberculosis patients, previous active TB infection, chest x-ray suggestive of tuberculosis, or a combination of the aforementioned. Second, both IGRA and TST should be used concurrently to identify LTBI within the same population. Third, provide the crucial data for analysis, encompassing true positives, false negatives, true negatives, and false positives, or provide data to calculate them.

We do not retrieve studies assessing only one of the two tests, IGRA or TST, conference abstracts, animal reports, or foreign language studies. The requirement for concurrent evaluation of both TST and IGRA within the same study population is essential to ensure a direct head-to-head comparison under identical clinical conditions, minimizing confounding from population-level differences in disease prevalence, immune status, and BCG vaccination rates. Studies evaluating only one test were excluded because they do not permit direct comparison of diagnostic performance between TST and IGRA, which is the primary objective of this meta-analysis. This methodological approach is consistent with best practices recommended for comparative diagnostic accuracy reviews.

### 2.2. Literature Search and Selection Process

A rigorous digital search was executed through several databases, including PubMed, Web of Science, Scopus, and Cochrane Library. Our search strategy was a mixture of several keywords related to tuberculous disease, tuberculin skin tests, and interferon-gamma release assays. A comprehensive search term for each database is presented in Supplementary Table S1.

The gathered articles were first imported into Endnote (Clarivate Analytics, Philadelphia, PA, USA) for the identification and removal of duplicate records. After deduplication, the remaining articles were assessed in two sequential screening phases. In the first phase, two reviewers independently evaluated articles based on their titles and abstracts and excluded those that clearly did not meet the inclusion criteria. In the second phase, the full texts of the remaining potentially eligible reports were retrieved and screened according to the predefined inclusion and exclusion criteria. Furthermore, a manual screening of the reference lists of previous meta-analyses and systematic reviews was executed to identify additional eligible studies. Any disagreements between the reviewers were resolved through discussion or consultation with a third reviewer.

### 2.3. Data Collection

A pre-designed data extraction sheet was implemented to gather the subsequent data: country, recruitment time, sample size, age category (Either adults, Children, or mixed), BCG vaccination, LTBI gold standard, IGRA type, TST cut-off values, and conclusion.

### 2.4. Quality Assessment

QUADAS-2 tool was employed to assess the quality of the included studies [13]. This tool assesses the quality of diagnostic studies regarding four main items: patient selection, index test methodology, reference standard characteristics, flow and timing process. Through this evaluation, both the risk of bias and applicability issues within these domains were appraised.

### 2.5. Data Analysis

OpenMeta Analyst software (version 0.24.1, released 20 September 2023) was employed to execute this diagnostic meta-analysis. A bivariate random-effects model was employed to jointly estimate pooled sensitivity and specificity while accounting for the inherent negative correlation between these two measures across studies. This approach is particularly appropriate given the potential threshold effects associated with different TST cut-off values. For subgroup analyses with fewer than four studies, where the bivariate model may not converge, univariate random-effects models were applied, and this is noted in the corresponding results. Sensitivity (SN) and specificity (SP) with their corresponding 95% confidence intervals (CI) for LTBI were synthesized for both TST and IGRA tests. The sensitivity is calculated as the proportion of true positive cases to the total of true positives and false negatives. Specificity was derived by dividing the number of true negatives by the aggregate total of true negatives and false positives. We grouped the analysis according to the age category, LTBI reference standard test, population type, IGRA type, and TST’s cut-off value. Significant heterogeneity was considered if the chi-square *p*-value < 0.1 or I-square was more than or equal to 50%.

## 3. Results

### 3.1. Search Results

**Figure 1:**
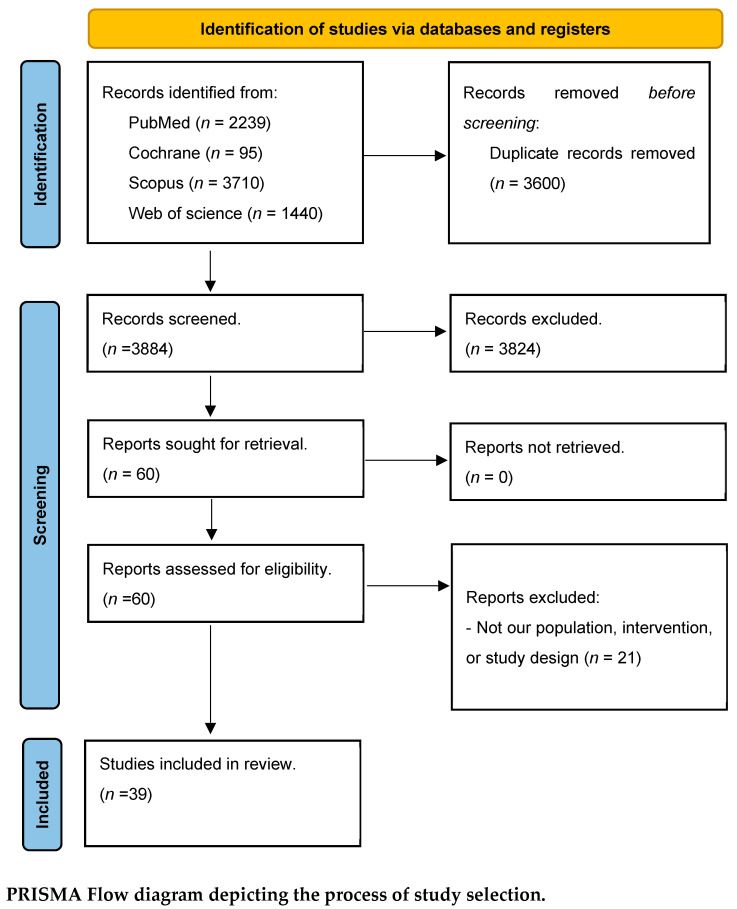
PRISMA Flow chart. Preferred Reporting Items for Systematic Reviews and Meta-Analyses (PRISMA) flow diagram illustrating the study selection process. A total of 7484 records were identified from database searches (PubMed, Web of Science, Scopus, and Cochrane Library). After removing 3600 duplicates, 3884 records underwent title and abstract screening, with 3824 excluded. Sixty full-text articles were assessed for eligibility, and 39 studies were finally included in the diagnostic meta-analysis.

Our primary digital search retrieved 7484 records; of them, 3600 articles were eliminated as duplicates utilizing Endnote and 3884 articles were initially screened based on their title and abstracts, excluding 3824 articles during this phase. Furthermore, 60 reports were eligible for the further screening phase, ending with 39 articles included in our diagnostic meta-analysis. Specifically, the initial database search across PubMed, Web of Science, Scopus, and Cochrane Library yielded a total of 7484 records. After importing all records into Endnote, 3600 duplicate records were identified and removed, leaving 3884 unique articles for screening. During the first screening phase (title and abstract review), 3824 articles were excluded as they did not meet the inclusion criteria, leaving 60 articles eligible for full-text assessment. Following detailed full-text evaluation against the predefined inclusion and exclusion criteria, 21 articles were further excluded, resulting in 39 studies that were finally included in the quantitative synthesis. The PRISMA flow diagram representing the study selection process is presented in Figure 1.

### 3.2. Characteristics of the Included Studies

Our diagnostic meta-analysis finally included thirty-nine studies [14,15,16,17,18,19,20,21,22,23,24,25,26,27,28,29,30,31,32,33,34,35,36,37,38,39,40,41,42,43,44,45,46,47,48,49,50,51,52]. The retrieved studies were conducted in several geographical regions: sixteen in Europe, fifteen in Asia, seven in North and South America, and one in Africa, particularly in Egypt. The majority of studies (21) were conducted in adult individuals, with 13 studies conducted in children. The most commonly utilized LTBI gold standard proxy was previous contact with active TB cases, with a total of 22 studies. Many IGRAs were employed across the included studies, with the QFT-GIT assay being the most commonly used test. A detailed summarization of the included studies is outlined in Supplementary Table S2. The conclusions reported in the Supplementary Table S2 represent verbatim extracts from the original studies and reflect each study’s individual interpretation rather than the inclusion criteria of the present meta-analysis.

### 3.3. Quality Assessment

**Figure 2:**
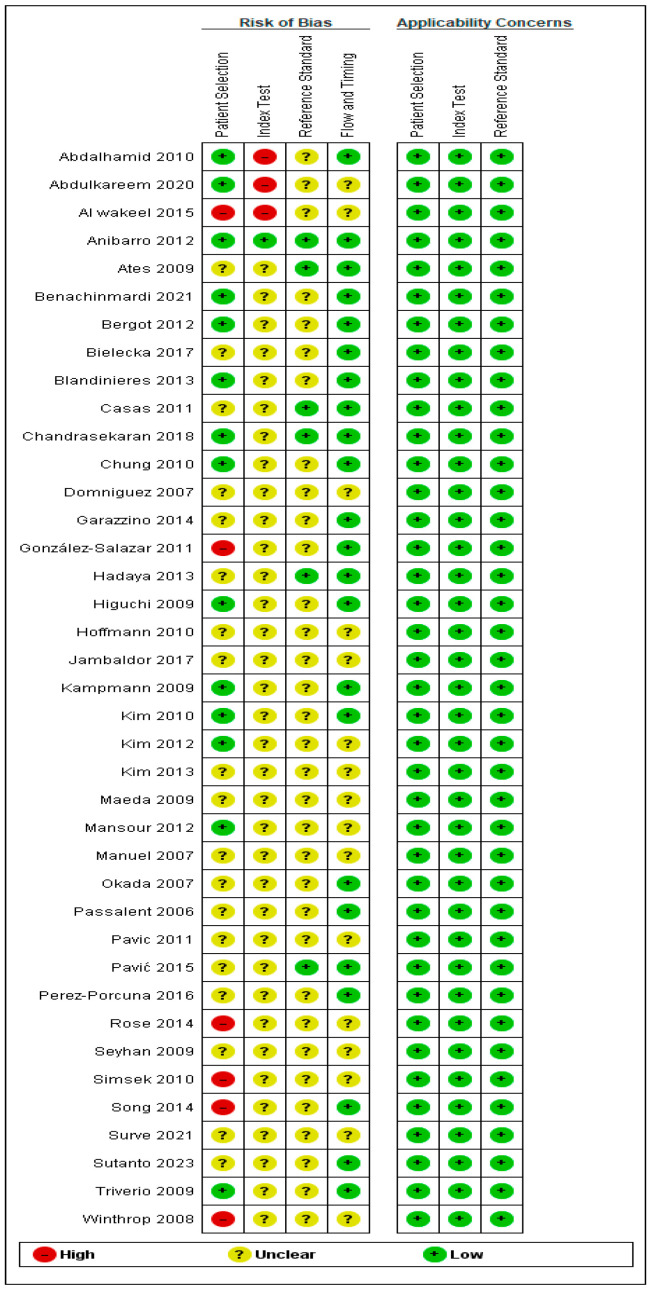
Quality assessment of the included studies. Summary of risk of bias and applicability concerns assessed using the Quality Assessment of Diagnostic Accuracy Studies (QUADAS-2) tool for each of the 39 included studies. The four assessed domains are patient selection, index test, reference standard, and flow and timing. Green indicates low risk, yellow indicates unclear risk, and red indicates high risk of bias [14,15,16,17,18,19,20,21,22,23,24,25,26,27,28,29,30,31,32,33,34,35,36,37,38,39,40,41,42,43,44,45,46,47,48,49,50,51,52].

Utilizing the QUADAS-2 assessment tool demonstrated that most of the included studies showed low concerns regarding the applicability domains, encompassing patient selection, index test, and reference standard. Furthermore, the patient selection domain was most frequently associated with unclear risk. Also, the index test domain revealed an unclear risk across the majority of included studies, with only six studies showing a high risk of bias. Specifically, the unclear risk in the patient selection domain was primarily attributable to insufficient reporting of consecutive or random enrollment of participants. The high risk of bias observed in the index test domain across six studies was mainly related to the lack of blinding in the interpretation of the TST or IGRA results. The flow and timing domain showed low risk in the majority of studies, indicating that most studies applied both index tests and the reference standard within an acceptable timeframe. Overall, while the applicability concerns were generally low, the risk of bias findings highlight the need for cautious interpretation of pooled diagnostic accuracy estimates. The detailed risk of bias for each involved study is outlined in Figure 2.

### 3.4. Diagnostic Accuracy Outcomes

#### 3.4.1. Overall Analysis

**Figure 3:**
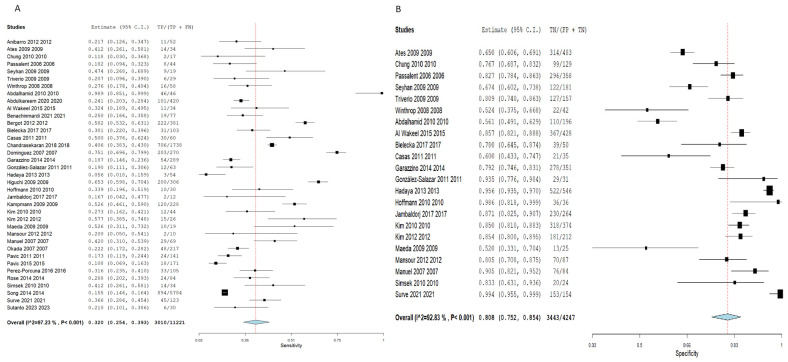
Forest plot of estimates of sensitivity and specificity for TB diagnosis via TST in all populations. Individual study estimates and the pooled sensitivity (SN = 0.320, 95% CI: 0.254–0.393) and specificity (SP = 0.808, 95% CI: 0.752–0.854) of the tuberculin skin test (TST) for the diagnosis of latent tuberculosis infection (LTBI) across all 39 included studies. Each square represents an individual study estimate, with horizontal lines indicating 95% confidence intervals. The diamond represents the pooled estimate. Substantial heterogeneity was observed (SN: I^2^ = 97.23%; SP: I^2^ = 92.83%). (**A**) Sensitivity forest plot; (**B**) Specificity forest plot [14,15,16,17,18,19,20,21,22,23,24,25,26,27,28,29,30,31,32,33,34,35,36,37,38,39,40,41,42,43,44,45,46,47,48,49,50,51,52].

**Figure 4:**
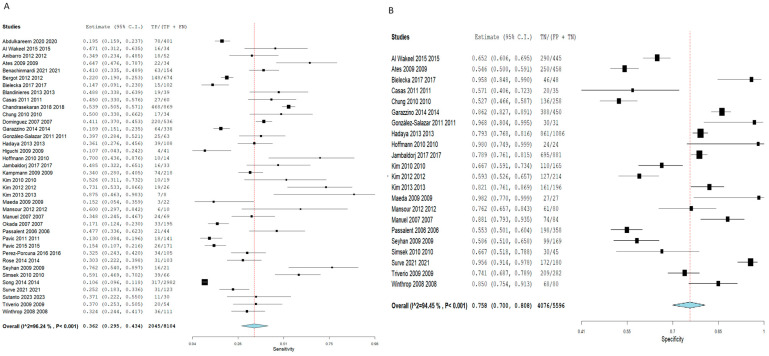
Forest plot of estimates of sensitivity and specificity for TB diagnosis via IGRA in all populations. Individual study estimates and the pooled sensitivity (SN = 0.362, 95% CI: 0.295–0.434) and specificity (SP = 0.758, 95% CI: 0.700–0.808) of the interferon-gamma release assay (IGRA) for LTBI diagnosis across all included studies. The 95% confidence intervals of pooled TST and IGRA sensitivity and specificity show considerable overlap, suggesting the difference between the two tests may not be statistically significant. Substantial heterogeneity was observed (SN: I^2^ = 96.24%; SP: I^2^ = 94.45%). (**A**) Sensitivity forest plot; (**B**) Specificity forest plot [14,15,16,17,18,19,20,21,22,23,24,25,26,27,28,29,30,31,32,33,34,35,36,37,38,39,40,41,42,43,44,45,46,47,48,49,50,51,52].

Employing the TST for the detection of latent TB cases demonstrated an SN of 0.320 (95% CI: 0.254–0.393), and an SP of 0.808 (95% CI: 0.752–0.854). Pooled studies were linked to a substantial heterogeneity (SN: I^2^ = 97.23%, *p* < 0.001, SP: I^2^ = 92.83%, *p* < 0.001), as illustrated in Figure 3. Nevertheless, employing the IGRA demonstrated a slightly elevated SN estimated at 0.362 (95% CI: 0.295–0.434) and a lower SP estimated at 0.758 (95% CI: 0.700–0.808) compared to TST, although the 95% confidence intervals overlapped considerably between the two tests, suggesting these differences may not be statistically significant. Pooled studies showed a significant heterogeneity (SN: I^2^ = 96.24%, *p* < 0.001, SP: I^2^ = 94.45%, *p* < 0.001), as illustrated in Figure 4.

#### 3.4.2. Based on Age

##### Adults

**Figure 5:**
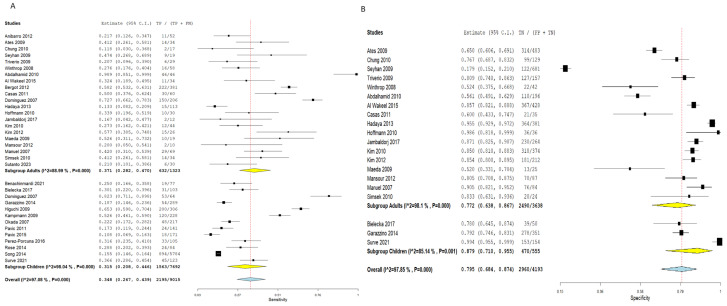
Forest plot of estimates of sensitivity and specificity for TB diagnosis via TST with subgroup analysis according to the age of the population. The forest plot displays stratified pooled estimates of TST sensitivity and specificity for adults (SN = 0.371; SP = 0.772) and children (SN = 0.315; SP = 0.879). Notably, in children, TST showed higher sensitivity and specificity compared to adults, although heterogeneity was significant in both subgroups. (**A**) Sensitivity forest plot; (**B**) Specificity forest plot [14,15,16,17,18,19,20,21,22,23,24,25,26,27,28,29,30,31,32,33,34,35,36,37,38,39,40,41,42,43,44,45,46,47,48,49,50,51,52].

Utilizing TST to diagnose adult latent TB cases revealed an SN of 0.371 (95% CI: 0.282–0.470) and an SP estimated at 0.772 (95% CI: 0.638–0.867). The combined results showed a significant heterogeneity (SN: I^2^ = 88.99%, *p* = 0.001, SP: I^2^ = 98.1%, *p* = 0.001), as outlined in Figure 5.

**Figure 6:**
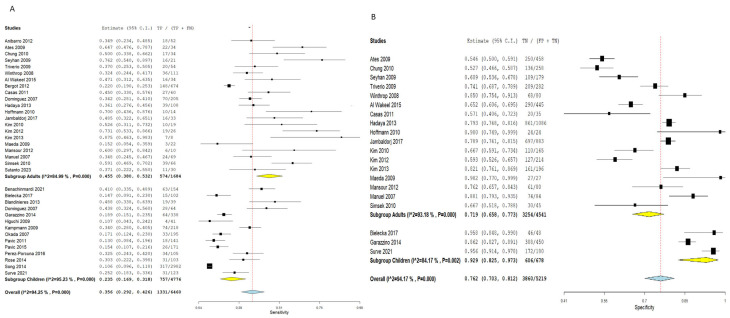
Forest plot of estimates of sensitivity and specificity for TB diagnosis via IGRA with subgroup analysis according to the age of the population. The forest plot displays stratified pooled IGRA estimates for adults (SN = 0.455; SP = 0.719) and children (SN = 0.235; SP = 0.929). In the pediatric population, IGRA demonstrated lower sensitivity but higher specificity compared to TST, potentially reflecting immature immune responses and lower BCG cross-reactivity. (**A**) Sensitivity forest plot; (**B**) Specificity forest plot [14,15,16,17,18,19,20,21,22,23,24,25,26,27,28,29,30,31,32,33,34,35,36,37,38,39,40,41,42,43,44,45,46,47,48,49,50,51,52].

Concerning IGRA for the detection of adult latent TB patients, the pooled analysis showed a higher SN estimated by 0.455 (95% CI: 0.380–0.532) and a lower specificity estimated by 0.719 (95% CI: 0.658–0.773) relative to TST. Pooled studies reveal a notable heterogeneity (SN: I^2^ = 84.99%, *p* = 0.001, SP: I^2^ = 93.18%, *p* = 0.001), as shown in Figure 6.

##### Children

TST for the diagnosis of latent TB within the children population yielded an SN of 0.315 (95% CI: 0.208–0.446) and an SP of 0.879 (95% CI: 0.710–0.955). The combined results showed significant heterogeneity (SN: I^2^ = 98.04%, *p* = 0.001, SP: I^2^ = 85.14%, *p* = 0.001), as pointed out in Figure 5. However, within the children population, the IGRA showed a lower SN estimated by 0.235 (95% CI: 0.169–0.318) and a higher SP estimated by 0.929 (0.825–0.973) compared to TST. The combined results were not homogeneous (SN: I^2^ = 95.23%, *p* = 0.001, SP: I^2^ = 84.17%, *p* = 0.002), as shown in Figure 6.

#### 3.4.3. Reference Standard Proxy

##### Previous Contact

**Figure 7:**
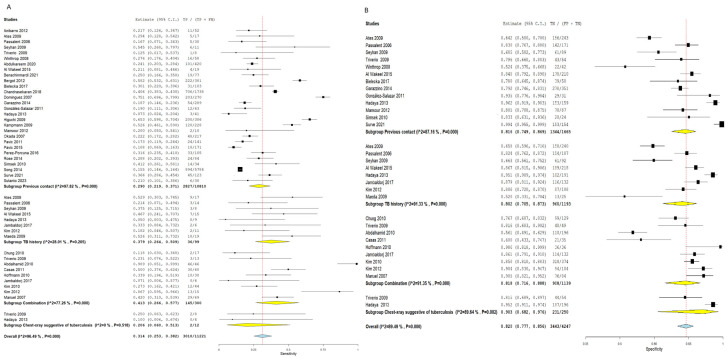
Forest plot of estimates of sensitivity and specificity for TB diagnosis via TST with subgroup analysis according to the gold standard proxy. Subgroups include previous contact, TB history, a combination of reference standards, and chest X-ray suggestive of tuberculosis. Across all proxy reference standards, TST consistently demonstrated lower sensitivity and higher specificity relative to IGRA. Heterogeneity was notable across most subgroups. (**A**) Sensitivity forest plot; (**B**) Specificity forest plot [14,15,16,17,18,19,20,21,22,23,24,25,26,27,28,29,30,31,32,33,34,35,36,37,38,39,40,41,42,43,44,45,46,47,48,49,50,51,52].

**Figure 8:**
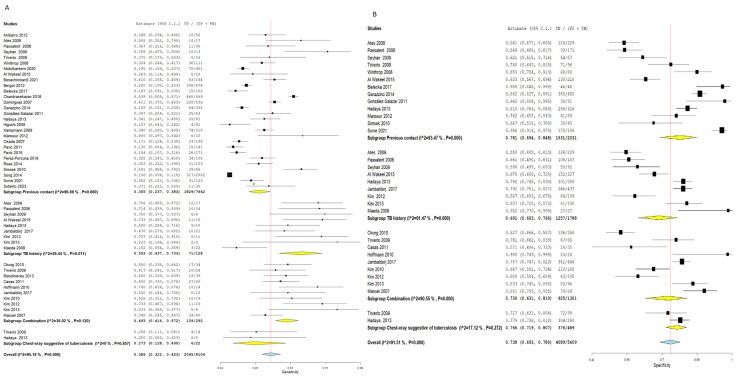
Forest plot of estimates of sensitivity and specificity for TB diagnosis via IGRA with subgroup analysis according to the gold standard proxy. Subgroups include previous contact, TB history, combination, and chest X-ray suggestive of TB. IGRA showed consistently higher sensitivity and lower specificity relative to TST across all proxy standards, with the most pronounced difference observed when TB history was used as the reference standard (IGRA SN = 0.593 vs. TST SN = 0.379). (**A**) Sensitivity forest plot; (**B**) Specificity forest plot [14,15,16,17,18,19,20,21,22,23,24,25,26,27,28,29,30,31,32,33,34,35,36,37,38,39,40,41,42,43,44,45,46,47,48,49,50,51,52].

Using the TST for the detection of latent TB patients with the previous contact as a standard proxy yielded an SN of 0.290 (95% CI:0.219–0.371) and an SP of 0.816 (95% CI: 0.749–0.869). The pooled results were linked to a notable heterogeneity (SN: I^2^ = 97.82%, *p* = 0.001, SP: I^2^ = 87.16%, *p* = 0.001), as illustrated in Figure 7. Interestingly, employing the IGRA for diagnosis of latent TB cases with the previous contact as a standard proxy showed a higher SN estimated by 0.305 (95% CI: 0.237–0.383) and a lower specificity estimated by 0.781 (95% CI: 0.694–0.848) compared to TST. The pooled results were heterogeneous (SN: I^2^ = 96.89%, *p* = 0.001, SP: I^2^ = 93.47%, *p* = 0.001), as shown in Figure 8.

##### TB History

Latent TB diagnosis by TST with the TB history as standard proxy revealed a pooled SN of 0.379 (95% CI: 0.264–0.509) and an SP of 0.802 (95% CI: 0.705–0.873). The overall studies were associated with notable heterogeneity regarding SP (I^2^ = 91.33%, *p* = 0.001) and a notable non-significant heterogeneity concerning SN (I^2^ = 28.01%, *p* = 0.205), as shown in Figure 7.

Furthermore, the IGRA exhibited a higher SN estimated by 0.593 (95% CI: 0.437–0.733) and a lower SP estimated by 0.691 (95% CI: 0.603–0.766). Pooled studies were heterogeneous (SN: I^2^ = 59.44%, *p* = 0.011, SP: I^2^ = 91.47%, *p* = 0.001), as illustrated in Figure 8.

##### Combination

Using the combination as a proxy, the TST showed a pooled SN of 0.413 (95% CI: 0.266–0.577) and SP of 0.818 (95% CI: 0.716–0.888). The pooled results were heterogeneous (SN: I^2^ = 77.26%, *p* = 0.001, SP: I^2^ = 91.35%, *p* = 0.001), as shown in Figure 7. Nevertheless, the IGRA revealed a higher SN estimated by 0.493 (95% CI: 0.416–0.572) and a lower SP estimated by 0.730 (95% CI: 0.631–0.810) when compared to TST. The pooled results were homogenous regarding SN (I^2^ = 36.02%, *p* = 120) and heterogeneous regarding SP (I^2^ = 90.5%, *p* = 0.001), as illustrated in Figure 8.

##### Chest X-Ray Suggestive of Tuberculosis

The TST for the detection of latent TB yielded an SN of 0.206 (95% CI: 0.060–0.513) and an SP of 0.903 (95% CI: 0.682–0.976). The overall results were homogenous concerning SN (I^2^ = 0%, *p* = 0.518) and heterogeneous regarding SP (I^2^ = 89.64%, *p* = 0.002), as illustrated in Figure 7.

Moreover, the IGRA showed a slightly higher SN estimated at 0.273 (95% CI: 0.128–0.490) and a lower SP estimated at 0.766 (95% CI: 0.719–0.807) compared to TST. The pooled studies were homogenous (SN: I^2^ = 0%, *p* = 0.857, SP: I^2^ = 17.12%, *p* = 0.272), as pointed out in Figure 8.

#### 3.4.4. Based on Population

##### Hemodialysis

Employing TST to diagnose latent TB cases within hemodialysis patients showed an SN of 0.278 (95% CI: 0.217–0.348) and SP of 0.754 (95% CI: 0.671–0.821). The pooled results were homogenous regarding SN (I^2^ = 36.4%, *p* = 0.127) and heterogenous regarding SP (I^2^ = 90.12%, *p* = 0.001), as presented in Supplementary Figure S1. Interestingly, IGRA revealed a higher SN estimated by 0.497 (95% CI: 0.397–0.596) and a lower SP estimated by 0.632 (95% CI: 0.559–0.700) compared to TST. The combined results were heterogeneous (SN: I^2^ = 71.1%, *p* = 0.001, SP: I^2^ = 88.76%, *p* = 0.001), as shown in Supplementary Figure S2.

##### Organ Transplant Patients

Latent TB diagnosis using TST within the organ transplant population showed an SN of 0.308 (95% CI: 0.187–0.462) and SP of 0.860 (95% CI: 0.788–0.911). Pooled studies showed a substantial heterogeneity (SN: I^2^ = 79.42%, *p* = 0.001, SP: I^2^ = 88.39%, *p* = 0.001), as illustrated in Supplementary Figure S1. Additionally, the IGRA revealed a higher SN estimated by 0.490 (95% CI: 0.389–0.593) and a lower SP estimated by 0.748 (95% CI: 0.683–0.803) compared to TST. The overall results were heterogeneous (SN: I^2^ = 63.08%, *p* = 0.008, SP: I^2^ = 0.89.34%, *p* = 0.001), as illustrated in Supplementary Figure S2.

#### 3.4.5. TST Cut-Off Value

Employing a TST with a cut-off value of 5 mm showed an SN of 0.329 (95% CI: 0.234–0.442) and an SP of 0.830 (95% CI: 0.747–0.889). The pooled results were heterogeneous (SN: I^2^ = 96.17%, *p* = 0.001, SP: I^2^ = 90.92%, *p* = 0.001), as illustrated in Supplementary Figure S3. Furthermore, TST with a cut-off value of 10 mm yielded a higher SN estimated by 0.342 (95% CI: 0.268–0.424) and a lower SP of 0.793 (95% CI: 0.710–0.856) compared to TST with a 5 mm cut-off value. Pooled studies revealed a notable heterogeneity (SN: I^2^ = 94.16%, *p* = 0.001, SP: I^2^ = 92.21%, *p* = 0.001), as shown in Supplementary Figure S3. Finally, the TST with a cut-off value of 15 mm revealed an SN of 0.231 (95% CI: 0.093–0.466) with heterogeneous pooled results (I^2^ = 97.7%, *p* = 0.001) and an SP of 0.833 (95% CI: 0.631–0.936).

#### 3.4.6. Based on the Type of IGRA

Utilizing QFT-GIT for the diagnosis of latent TB cases, the pooled SN was estimated at 0.363 (95% CI: 0.284–0.449), and the pooled SP was 0.764 (95% CI: 0.704–0.815). The pooled studies were heterogeneous (SN: I^2^ = 96.48%, *p* = 0.001, SP: I^2^ = 92.44%, *p* = 0.001), as presented in Supplementary Figure S4. However, the T. SPOT.TB exhibited a higher SN estimated by 0.420 (95% CI: 0.370–0.471) and a lower SP estimated by 0.671 (95% CI: 0.544–0.777) compared to QFT-GIT. Pooled studies showed substantial heterogeneity regarding SP (I^2^ = 94.29%, *p* = 0.001) and were not heterogeneous concerning SN (I^2^ = 27.23%, *p* = 0.193), as illustrated in Supplementary Figure S4.

## 4. Discussion

Our diagnostic meta-analysis encompassed 39 studies comparing the diagnostic utility of TST and IGRA for detecting LTBI. In the overall analysis, TST demonstrated marginally higher specificity but lower sensitivity compared to IGRA, though notably, the confidence intervals overlapped substantially in most comparisons. These patterns were generally consistent across adult populations and across all proxy reference standards, while the pediatric population exhibited a reversed pattern with TST showing higher sensitivity. The mechanistic basis for these observed differences in diagnostic performance merits discussion. The TST detects a delayed-type hypersensitivity response to purified protein derivative (PPD), a mixture of mycobacterial antigens, some of which are shared with BCG vaccine strains and non-tuberculous mycobacteria (NTM). This antigenic cross-reactivity accounts for the generally higher specificity of TST in non-BCG-vaccinated populations but may reduce specificity in BCG-vaccinated individuals. Conversely, IGRAs measure interferon-gamma released by T cells in response to M. tuberculosis-specific antigens (ESAT-6 and CFP-10), which are absent in BCG strains and most NTM species. This specificity to M. tuberculosis antigens renders IGRAs largely unaffected by BCG vaccination status but may result in lower sensitivity in immunocompromised patients whose T cell responses are attenuated. These fundamental immunological differences underpin the observed diagnostic performance patterns across different populations and clinical settings.

Without a definitive reference test for diagnosing LTBI, the TST and IGRAs are employed to aid in detecting LTBI and to guide clinical decisions, mainly in high-income nations [53]. However, the available literature reports conflicting results between the two tests regarding the diagnosis of LTBI. With an application of strict eligibility criteria, in which we only include studies evaluating TST and IGRA within the same population with a reference gold standard proxy, encompassing previous contact with an active TB patient, history of TB disease, chest-x-ray suggestive of TB, or a combination of the aforementioned, we aimed to update the available evidence and provide the clinicians with the most suitable and effective method for diagnosis of LTBI. In their diagnostic meta-analysis, Ferguson and his colleagues investigated the diagnostic performance of TST and IGRAs in diagnosing LTBI within the hemodialysis population [4]. With seventeen studies finally included, they concluded that TST was associated with lower SN compared to IGRAs, including QFT-GIT and T. SPOT.TB was aligned with our overall and grouped analyses within hemodialysis patients only. Current clinical guidelines recommend using TST to determine LTBI in hemodialysis and other immunosuppressed patients, possibly according to cost-effectiveness estimates derived from the general population [54,55,56]. However, these estimates may be unreliable for hemodialysis patients for several reasons. Primarily, the higher prevalence of anergy in these patients mitigates the diagnostic utility of TST [31,41]. Secondly, the risk of reactivation to active communicable disease in this population may be markedly increased, especially after organ transplantation, indicating that lower sensitivity could have substantial clinical consequences not present in the general population [57].

Nevertheless, Ferguson et al. also found that TST was linked to a lower SP than IGRA, which does not agree with our findings. This finding could be attributed to the fact that in their methodology, they included studies that assessed both tests, TST and IGRA, for diagnosis of latent TB infection, eventually within the same population or even one test of the two tests alone within a different population, which could potentially overestimate their effect estimates. Furthermore, they also employed a gold standard proxy as a reference test. When they employed the previous contact with TB patients as the reference standard, they found that TST was correlated to a lower SN but higher SP relative to IGRA, especially QFT-GIT. Our results aligned with them as we found that when the previous contact was used as a reference standard, the TST showed lower SN and higher SP than IGRA. Moreover, when they utilized the history of tuberculosis disease as a reference standard, they found a lower SN with TST relative to IGRA, with an equal SP between the two tests. A previous prospective study conducted by Korra and his colleagues examined the diagnostic utility of IGRA, especially QFT-GIT, compared to TST for detecting LTBI within immunocompromised patients [58]. Their findings were consistent with ours, as they found that IGRA was associated with higher SN than TST. Adams et al. [59] also investigated the diagnostic utility of TST and IGRAs for diagnosing LTBI utilizing a latent class analysis. Their results were inconsistent with ours, as they concluded that TST was linked to a higher SN and lower SP than IGRAs. However, their findings are limited because they conducted their study in the country’s high TB endemic setting. Also, most of their included population was vaccinated with BCG, potentially raising the chance for positive results within TST. Additionally, our results were consistent with those of Al Wakeel et al., who found that TST was correlated to a lower SN and a higher SP relative to IGRA [16].

Furthermore, our results within the pediatric population showed that TST was linked to a higher SN and a lower SP than IGRA, which is inconsistent with the other results. This could be explained by the fact that young children are known for their immature immune systems compared to adults. As a result, TB-specific interferon-gamma-producing cells might be present in lower numbers in children than in adults. These lower immune responses result in lower interferon-gamma values, the specific cytokine measured in IGRAs, and eventually lower SN and higher SP results [33].

An important consideration in interpreting the higher TST sensitivity observed in children is the high prevalence of BCG vaccination among the included pediatric populations. BCG vaccination, typically administered at birth or in early childhood in many of the study countries, may have contributed to cross-reactivity with TST antigens, leading to false-positive results that inflate apparent sensitivity. This BCG-related cross-reactivity may partly explain the lower specificity of TST relative to IGRA in children. In contrast, IGRAs utilize M. tuberculosis-specific antigens (ESAT-6 and CFP-10) that are absent in BCG strains, making them less susceptible to BCG-induced cross-reactivity. Future studies should systematically stratify pediatric results by BCG vaccination status to better delineate the true diagnostic performance of each test in children.

The diagnostic performance of TST and IGRAs may also be influenced by demographic diversity within the studied populations, including sex-related immunological differences. However, the majority of the included studies did not provide sex-stratified data, representing a notable gap in the current literature and limiting our ability to draw conclusions regarding this important dimension. Future studies should aim to systematically stratify results by sex, age group, and comorbidity status to enable more precise diagnostic recommendations tailored to specific population subgroups.

Furthermore, the seemingly counterintuitive finding that the 10 mm TST cut-off yielded higher sensitivity than the 5 mm cut-off warrants careful interpretation. This finding is likely attributable to compositional differences between the study populations in each subgroup rather than a true threshold effect. The studies employing the 5 mm cut-off included a higher proportion of immunocompromised patients (e.g., hemodialysis and organ transplant recipients), in whom attenuated immune responses may have reduced TST sensitivity regardless of the threshold used. Therefore, this finding should not be interpreted as evidence that a 10 mm cut-off is inherently more sensitive than a 5 mm cut-off. Rather than recommending a single universal TST threshold, clinicians should consider the patient’s individual risk profile, including immunosuppression status, BCG vaccination history, and pre-test probability of LTBI, when selecting the most appropriate cut-off value, in accordance with national and WHO guidelines [60,61,62,63].

Our diagnostic meta-analysis possesses several strengths. First, to our best knowledge, our study provides the most updated and comprehensive evidence regarding the diagnostic utility of TST compared to IGRA for the detection of LTBI. Second, the included studies were conducted in several geographical regions with different population demographics that support the generalizability of our findings to the general population. Third, the strict eligibility criteria of our study with several subgroups could also provide a piece of more robust evidence regarding the differences in the diagnostic performance between TST and IGRAs. Nevertheless, our study also contains several limitations. First, the retrospective nature of the majority of the included studies potentially introduces some selection bias and affects the validity and consistency of our results. Second, the observed heterogeneity across most outcomes could also affect the validity and generalizability of our findings. Different populations, geographical regions, detection thresholds, and procedural variations across the included studies could explain this heterogeneity. Third, the absence of a definitive gold standard for LTBI diagnosis represents a fundamental limitation inherent to all diagnostic accuracy studies in this field. The reliance on proxy reference standards, such as previous TB contact, history of TB disease, or radiographic findings, may introduce misclassification bias that could either overestimate or underestimate the true diagnostic accuracy of both TST and IGRA. Fourth, it is important to acknowledge that in the majority of our analyses, the differences in pooled sensitivity and specificity between TST and IGRA were modest, with substantial overlap in the 95% confidence intervals. Therefore, these findings do not definitively establish the superiority of one test over the other, and the clinical significance of these numerical differences may be limited. Fifth, the majority of included studies did not report sex-stratified data, precluding analysis of potential sex-related differences in diagnostic performance.

Our diagnostic meta-analysis provides crucial insights for clinicians regarding the diagnosis of LTBI. Our primary analysis showed that TST generally exhibits higher SP but lower SN relative to IGRA. This is consistent across several subgroups, encompassing adults, hemodialysis, and organ transplant patients. However, notable differences were noted in the pediatric population, where TST shows higher sensitivity and lower specificity. In clinical settings, these findings recommend that TST could be a preferable option in certain settings owing to its high specificity and its lower sensitivity compared to IGRAs, which should be crucially considered, particularly within immunocompromised populations, encompassing HIV and hemodialysis patients, where the risk of reactivation to active disease is notable. Furthermore, IGRAs have a higher sensitivity, especially T. SPOT.TB may offer more reliable detection in these high-risk groups. Additionally, our findings highlight that the diagnostic performance of TST can vary depending on the cut-off value utilized. A 10 mm cut-off provides higher sensitivity but lower specificity than a 5 mm cut-off, suggesting clinicians need to optimize the threshold according to the specific clinical scenario and patient risk factors.

Given the significant heterogeneity and the limitations observed within the included studies, future research should aim to standardize methodologies to improve comparability and reliability. Prospective studies with rigorous designs are necessary to reduce the selection bias associated with retrospective studies and to validate our findings across diverse populations.

Moreover, additional research should focus on the long-term outcomes of patients diagnosed with LTBI using TST versus IGRAs, particularly in immunocompromised populations such as those undergoing hemodialysis or organ transplantation. Studies evaluating the cost-effectiveness of each diagnostic method in these high-risk groups could provide further insights to optimize clinical guidelines. Investigation into the impact of BCG vaccination on TST performance is also needed, especially in high-TB-endemic settings. This would help delineate the contexts in which TST might produce false positives and inform the development of more accurate diagnostic algorithms.

## 5. Conclusions

Our diagnostic meta-analysis suggests that TST and IGRA may have comparable overall diagnostic performance for LTBI, with TST exhibiting marginally higher specificity and IGRA showing marginally higher sensitivity. However, given the substantial overlap in confidence intervals and the high heterogeneity across the included studies, these differences should be interpreted with caution, and neither test can be definitively deemed superior to the other. Important subgroup differences were observed: in pediatric populations, TST demonstrated higher sensitivity but lower specificity, potentially influenced by BCG vaccination-related cross-reactivity, and in immunocompromised populations such as hemodialysis and organ transplant patients, the lower sensitivity of TST may have significant clinical implications. The reliance on proxy reference standards in the absence of a true gold standard for LTBI further limits the certainty of these estimates. The findings advocate for a tailored, patient-centered approach to LTBI diagnosis, considering individual risk factors, BCG vaccination history, immune status, and the local epidemiological context. Future prospective studies employing standardized methodologies, bivariate analytical approaches, and sex- and age-stratified analyses are warranted to validate these findings and guide the development of optimized diagnostic algorithms for specific population subgroups.

## Data Availability

The original contributions presented in this study are included in the article/Supplementary Materials. Further inquiries can be directed to the corresponding author.

## Supplementary Materials

The following supporting information can be downloaded at https://www.mdpi.com/article/10.3390/diagnostics16060951/s1, Prisma 2020 Checklist; Supplementary Figure S1: Forest plot of estimates of sensitivity and specificity for TB diagnosis via TST with subgroup analysis according to the population; Supplementary Figure S2: Forest plot of estimates of sensitivity and specificity for TB diagnosis via IGRA with subgroup analysis according to the population; Supplementary Figure S3: Forest plot of estimates of sensitivity and specificity for TB diagnosis via TST with subgroup analysis according to TST cut-off values; Supplementary Figure S4: Forest plot of estimates of sensitivity and specificity for TB diagnosis via IGRA with subgroup analysis according to the IGRA types; Supplementary Table S1: Detailed search strategy for the searched databases; Supplementary Table S2: Summary features of the included studies (previously Supplementary Table S1, relocated to supplementary materials per reviewer recommendation). Detailed search strategy for the searched databases.
