# Supplementary material for: Comparative Diagnostic Performance of TST and IGRAs in the Diagnosis of Latent Tuberculosis Infection: A Systematic Review and Diagnostic Meta-Analysis
Source: Diagnostics (Basel). 2026 Mar 23;16(6):951. doi: 10.3390/diagnostics16060951 (PMC13025171; doi:10.3390/diagnostics16060951)
Supplement: Supplementary file 1 [file diagnostics-16-00951-s001.zip › Supplementary Table S1.pdf]

Supplementary Table S1: Detailed search strategy for the searched databases

**Appendix A**: databases were used to search for articles related to the following key words:		
**Databases**	**Search Strategy**	**Results**
**Pubmed**	("QFT" OR "T-SPOT" OR "SPOT" OR "Interferon-gamma Release Test*" OR "Release Test*, Interferon-gamma" OR "Test*, Interferon-gamma Release" OR "Interferon-gamma Release Assay*" OR "Interferon gamma Release Assay*" OR "Assay*, Interferon-gamma Release" OR "Interferon gamma Release Assay" OR "Release Assay*, Interferon-gamma" OR "IGRA") AND<br>("Tuberculin Test" OR "Test, Tuberculin" OR "Tests, Tuberculin" OR "Tuberculin Tests" OR "TST" OR "PPD-B" OR "PPD B" OR "PPD-L" OR "PPD L" OR "Purified Protein Derivative of Tuberculin" OR "PPD" OR "PPD-S" OR "PPD-S" OR "PPD-CG" OR "PPD CG" OR "PPD-F" OR "PPD F") AND<br>("Tuberculosis" OR "Tuberculoses" OR "Kochs Disease" OR "Koch's Disease" OR "Koch Disease" OR "Infection*, Mycobacterium tuberculosis" OR "Mycobacterium tuberculosis Infection*")	2339
**Scopus**	TITLE-ABS-KEY (("qft" OR "t-spot" OR "spot" OR "interferon-gamma release test*" OR "release test*, interferon-gamma" OR "test*, interferon-gamma release" OR "interferon-gamma release assay*" OR "interferon gamma release assay*" OR "assay*, interferon-gamma release" OR "interferon gamma release assay" OR "release assay*, interferon-gamma" OR "igra") AND ("tuberculin test" OR "test, tuberculin" OR "tests, tuberculin" OR "tuberculin tests" OR "tst" OR "ppd-b" OR "ppd b" OR "ppd-l" OR "ppd l" OR "purified protein derivative of tuberculin" OR "ppd" OR "ppd-s" OR "ppd-s" OR "ppd-cg" OR "ppd cg" OR "ppd-f" OR "ppd f") AND ("tuberculosis" OR "tuberculoses" OR "kochs disease" OR "koch's disease" OR "koch disease" OR "infection*, mycobacterium tuberculosis" OR "mycobacterium tuberculosis infection*"))	3710

**Web of science**	TS=("QFT" OR "T-SPOT" OR "SPOT" OR "Interferon-gamma Release Test*" OR "Release Test*, Interferon-gamma" OR "Test*, Interferon-gamma Release" OR "Interferon-gamma Release Assay*" OR "Interferon gamma Release Assay*" OR "Assay*, Interferon-gamma Release" OR "Interferon-gamma Release Assay" OR "Release Assay*, Interferon-gamma" OR IGRA) AND<br>TS=("Tuberculin Test" OR "Test, Tuberculin" OR "Tests, Tuberculin" OR "Tuberculin Tests" OR "TST" OR "PPD-B" OR "PPD B" OR "PPD-L" OR "PPD L" OR "Purified Protein Derivative of Tuberculin" OR "PPD" OR "PPD-S" OR "PPD-S" OR "PPD-CG" OR "PPD CG" OR "PPD-F" OR "PPD F") AND<br>TS=("Tuberculosis" OR "Tuberculoses" OR "Kochs Disease" OR "Koch's Disease" OR "Koch Disease" OR "Infection*, Mycobacterium tuberculosis" OR "Mycobacterium tuberculosis Infection*")	1440
**Cochrane library**	"QFT" OR "T-SPOT" OR "SPOT" OR "Interferon-gamma Release Test*" OR "Release Test*, Interferon-gamma" OR "Test*, Interferon-gamma Release" OR "Interferon-gamma Release Assay*" OR "Interferon gamma Release Assay*" OR "Assay*, Interferon-gamma Release" OR "Interferon gamma Release Assay" OR "Release Assay*, Interferon-gamma" OR "IGRA") AND ("Tuberculin Test" OR "Test, Tuberculin" OR "Tests, Tuberculin" OR "Tuberculin Tests" OR "TST" OR "PPD-B" OR "PPD B" OR "PPD-L" OR "PPD L" OR "Purified Protein Derivative of Tuberculin" OR "PPD" OR "PPD-S" OR "PPD-S" OR "PPD-CG" OR "PPD CG" OR "PPD-F" OR "PPD F") AND ("Tuberculosis" OR "Tuberculoses" OR "Kochs Disease" OR "Koch's Disease" OR "Koch Disease" OR "Infection*, Mycobacterium tuberculosis" OR "Mycobacterium tuberculosis Infection*") in Title Abstract Keyword - (Word variations have been searched)	95
**The total from the four databases:**		7484
**Number of duplicates:**		3600
**Number after removing duplication:**<br>**(By Endnote):**		3884
