# Supplementary material for: Comparative Diagnostic Performance of TST and IGRAs in the Diagnosis of Latent Tuberculosis Infection: A Systematic Review and Diagnostic Meta-Analysis
Source: Diagnostics (Basel). 2026 Mar 23;16(6):951. doi: 10.3390/diagnostics16060951 (PMC13025171; doi:10.3390/diagnostics16060951)
Supplement: Supplementary file 1 [file diagnostics-16-00951-s001.zip › Supplementary Table S2.pdf]

Supplementary Table S2. Summary features of the included studies.

Abbreviations: N: number, LTBI: latent tuberculosis infection, IGRA: Interferon-gamma release assays, QFT-GIT: QuantiFERON Gold in tube, TST: Tuberculin skin test,.

Study ID	Country	Recruitment Time	Sample Size	BCG Vaccination, N (%)	Age Category	LTBI Gold Standard	IGRA types	TST Cut-Off Values	Population Type	Conclusion
Abdalhamid 2010 [14]	USA	2006–2008	242	-	Adults	Combination	QFT-GIT	10 mm	Not specified	"A cost analysis of combining a tuberculin skin test (TST) and the QuantiFERON-TB Gold test (QFT-GT) to detect latent tuberculosis in newly hired healthcare workers was performed. An approximately 50% reduction in the cost of additional care was realized when workers with positive TST results were subsequently screened using the QFT-G."
Abdulkareem 2020 [15]	Iraq	May 2018–October 2018	521	498 (95.6%)	Mixed	Previous contact	QFT-plus	5 mm	Not specified	"The overall rate of LTBI was intermediate. Screening of LTBI should be routine among HHCs, regardless of the site of the disease. Age 15 years, alcoholics, immunosuppressive therapy, and PTB were potential risk factors. There was a good concordance between TST and QFT-Plus. A QFT-Plus can overcome the limitation of a BCG-vaccinated individual, especially in early life."
Al Wakeel 2015 [16]	Saudi Arabia	2011–2013	243	103 (42%)	Adults	Previous contact—TB history	QFT-GIT	Not specified	Peritoneal dialysis—hemodialysis	"Due to the high variability of QFT-GIT sensitivity, we recommend its use for its NPV and the use of either TST or QFT in screening latent TB."
Anibarro 2012 [17]	Spain	-	52	7 (13.5%)	Adults	Previous contact	QFT-GIT	5 mm	Hemodialysis	"In patients with ESRD, erythema without induration in the TST response could potentially be an indicator of M. tuberculosis infection. The QFT shows better accuracy for LTBI diagnosis than the TST."
Ates 2009 [18]	Turkey	March 2008–April 2008	275	108 (43%)	Adults	Previous contact—TB history	QFT-GIT	10 mm	Hemodialysis	"The present results suggest that the QTF-GIT is more sensitive than TST in the detection of LTBI among renal dialysis patients. Nevertheless, large longitudinal studies are required for more accurate results."
Benachinmardi 2021 [19]	India	-	77	-	Children	Previous contact	QFT-plus—TBF (TB-Fero)	5 mm	Not specified	"Hence, TBF, which showed similar efficiency as the widely used QFT Plus, can be a useful detection technique for LTBI in children. Moreover, it could prove to be an efficient alternative to expensive IGRAs like QFT Plus."
Bergot 2012 [20]	France	2007–2009	687	140 (20.4%)	Adults	Previous contact	QFT-GIT	10 mm	Not specified	"Our results confirm the safety of the QFT-based strategy for assessing the TB chemoprophylaxis indication, as only one contact developed TB disease out of 526 QFT-negative subjects."

Bielecka 2018 [21]	Poland	2009–2012	153	152 (99.3%)	Children	Previous contact	QFT-GIT	10 mm	Not specified	"The youngest children release a sufficient amount of IFN-γ in response to TB antigens; thus QFT-GIT might be a useful tool for TB diagnostics in this age group."
Blandinières 2013 [22]	France	2007–2011	226	72 (60%)	Children	Combination	QFT-GIT	Not specified	Immunocompetent	"In our low burden country, i) QF-TB-IT specificity was 100%, ii) QF-TB-IT sensitivity was low in infants but commensurable to adult values in older children, and iii) indeterminate results mostly relied on ongoing infections unrelated to TB."
Casas 2011 [23]	Spain	2008–2010	95	30 (31.6%)	Adults	Combination	QFT-GIT	5 mm	Liver transplantation candidates	"The QFT-IT test is better than the TST for detecting latent TB infection in patients with more advanced liver disease. Our results support the regular use of the QFT-IT test for screening patients with end-stage liver disease for latent TB infection."
Chandrasekaran 2018 [24]	India	-	869	483 (55%)	Mixed	Previous contact	QFT-GIT	5 mm–10 mm	Not specified	"Prevalence of LTBI among HHCs of adult pulmonary TB patients in India is very high and varies by test type, age, and exposure gradient. In our high TB burden setting, a strategy to treat all HHCs or a targeted strategy whereby an exposure index is used should be assessed in future preventive therapy and vaccine studies, as HHCs have several factors that place them at high risk for progression to TB disease."
Chung 2010 [25]	South Korea	March 2008–April 2008	167	111 (66%)	Adults	Combination	QFT-GIT-T. SPOT	10 mm	Hemodialysis	"The IGRAs can be useful for the diagnosis of latent TB infection in hemodialysis patients."
Domínguez 2008 [26]	Spain	2004–2006	626	274 (43%)	Mixed	Previous contact	QFT-GIT T. SPOT	5 mm	Not specified	"T-SPOT.TB was more sensitive than QFN-G-IT in diagnosing both active and latent infection. Both gamma interferon tests were unaffected by prior Mycobacterium bovis BCG vaccination. Among children who were not BCG vaccinated but had a positive tuberculin skin test, QFN-G-IT was negative in 53.3% of cases, and T-SPOT.TB was negative in 50% of cases."
Garazzino 2014 [27]	Italy	2005–2012	823	218 (26.5%)	Children	Previous contact	QFT-GIT	5 mm	Not specified	"QTF-IT showed good sensitivity and specificity, and a low rate of indeterminate results in the first 2 years of life, supporting its use at this age. However, considering costs and the similar performance between QTFIT and TST, it is reasonable to suggest the latter as first-line testing in young children. The complementary use of TST and interferon-γ release assays may be considered in selected cases to improve the accuracy of testing."
González-Salazar 2011 [28]	Mexico	January 2011–March 2011	106	98 (92.4%)	Mixed	Previous contact	QFT-GIT	5 mm	Not specified	"Our preliminary results show that the QTB-GIT has better capacity than TST to detect latent tuberculosis infection."
Hadaya 2013 [29]	Switzerland	2009–2011	200	155 (77.5%)	Adults	Previous contact—TB	QFT-GIT-T.SPOT	5 mm	Kidney transplantati	"Because their sensitivity for detecting prior active TB and probable LTBI in RTRs is very low, IGRAs cannot be used

						history—Chestx-ray suggestive of tuberculosis			on recipients	to exclude LTBI. These results emphasize the limitations of IGRAs in the setting of chronic immunosuppressive therapy."
Higuchi 2009 [30]	Japan	2005–2006	313	308 (98.4%)	Children	Previous contact	QFT-GIT	5 mm	Not specified	"These data suggest that QFT-G has the same performance characteristics in BCG-vaccinated children as it does in adults. The observation that none of the 297 students who were QFT-G negative had developed active TB after 3 years of follow-up suggests that QFT-G has a very high negative predictive value."
Hoffmann 2010 [31]	Switzerland	-	39	18 (46.1%)	Adults	Combination	QFT-GIT	5 mm–10 mm	Hemodialysis	"We conclude that the QFT-GIT is a valid alternative to the TST. Together with the survey of TB risk factors, it may help to diagnose LTBI more accurately in HD patients."
Jambaldorj 2017 [32]	South Korea	2009–2015	458	-	Adults	Combination—TB history	QFT-GIT	10 mm	Kidney transplantation recipients	"The QFT of recipients or living donors pre-KT cannot predict the short-term development of post-KT TB in an intermediate TB-burden country."
Kampmann 2009 [33]	United Kingdom	2006–2008	209	142 (67%)	Children	Previous contact	QFT-GIT-T.SPOT	15 mm–10 mm	Not specified	"A negative interferon-c release assay should not dissuade pediatricians from diagnosing and treating presumed active tuberculosis. If used for diagnosis of latent tuberculosis infection, interferon-c release assays could significantly reduce the number of children receiving chemoprophylaxis. Very good concordance between both tests was found."
Kim 2010 [34]	South Korea	2008–2009	209	145 (69%)	Adults	Combination	T.SPOT	5 mm–10 mm	Kidney transplantation candidates	"T-SPOT.TB test was more frequently positive than TST in renal transplant candidates. However, further longitudinal studies are awaited to determine whether the ability of T-SPOT.TB assay to detect LTBI in renal transplant recipients can better predict the development of TB than after transplantation."
Kim 2013 [35]	South Korea	2010–2012	126	115 (91.3%)	Adults	Combination—TB history	QFT-GIT	10 mm	Kidney transplantation candidates	"The positivity for QFT-GIT was higher than the positivity for TST, and QFT-GIT more accurately reflected the risk for LTBI. However, a further longitudinal study is needed in order to confirm that the QFT-GIT test can truly predict the development of TB after renal transplantation."
Kim 2013 [36]	South Korea	2008–2012	109	-	Adults	Combination—TB history	QFT-GIT	Not specified	Liver transplantation candidates	"QFT-GIT and TST for diagnosis of LTBI in KTRs showed reasonable concordance but no superiority of either test."
Maeda 2010 [37]	Japan	2005–2008	49	-	Adults	TB history	QFT-GIT	10 mm	Rheumatoid arthritis	"Using ROC analysis, the area under the curve (AUC) of QFT-G but not for the other two tests was significantly large. QFT-G is a useful diagnostic method due to its

										superior specificity, but the use of a cutoff value of 0.35 IU/mL will likely result in an underestimate. We propose that a lower interferon-c (IFN-c) titer of 0.1 IU/mL be adopted when deciding to administer anti-TB drugs before initiation of TNF inhibitors.”
Mansour 2012 [38]	Egypt	-	97	-	Adults	Previous contact	QFT-GIT	10 mm	Liver transplantation candidates	“This study showed that the QFT-GIT test might be more useful for the diagnosis of LTBI than TST among LTC on the basis of the frequency of clinical risk factors.”
Manuel 2007 [39]	Canada	2006–2007	153	116 (75.8%)	Adults	Combination	QFT-GIT	5 mm	Kidney transplantation candidates	“In patients awaiting liver transplantation, both the TST and QFT-G were comparable for the diagnosis of LTBI with reasonable concordance between tests. Indeterminate QFT-G result was more likely in those with more advanced liver disease.”
Okada 2008 [40]	Cambodia	-	217	191 (88%)	Children	Previous contact	QFT-GIT	10 mm	Not specified	“QFT can be a substitute for TST in detecting latent TB infection in childhood contacts aged >5 years, especially in those who may have a false-positive TST due to BCG vaccination or non-tuberculous mycobacterial infection.”
Passalent 2007 [41]	Canada	January 2005–April 2005	203	-	Not specified	Previous contact—TB history	T.SPOT	5 mm	Hemodialysis	“The TST is insensitive in hemodialysis patients and is not recommended to be used in isolation to diagnose latent tuberculosis infection. It is suggested that a combination of T-SPOT.TB testing and medical assessment may be the most accurate screening method.”
Pavić 2011 [42]	Croatia	2008–2009	142	-	Children	Previous contact	QFT-GIT	10 mm	Not specified	“Association of positive QFT and TST results with risk factors for infection in child contacts (presence of cavitary lesions and acid-fast bacilli smear positivity in index cases) suggests that both the tests have good diagnostic accuracy. However, there was significant discord between the results of the 2 tests that could not be definitively resolved. Thus, in a high-risk population of children up to 5 years of age, both tests (QFT and TST) should be performed, and the child should be considered infected if either or both tests are positive.”
Pavić 2015 [43]	Croatia	2010–2012	171	169 (98.8%)	Children	Previous contact	QFT-GIT	15 mm	Not specified	“The reasons for discordant results in young children are still unclear, which highlights the importance of further longitudinal studies to better understand the interpretation and any possible clinical implications of the results of these tests.”
Perez-Porcuna 2016 [44]	Brazil	2009–2010	121	-	Children	Previous contact	QFT-GIT	10 mm	Not specified	“This is one of the first studies to estimate the prevalence of LTBI in children and the parameters of the main diagnostic tests using a latent class model. Our results suggest that children in contact with an index case have a high risk of infection. The accuracy and the predictive value of the two tests did not significantly differ.

										Combined use of the two tests showed scarce improvement in the diagnosis of LTBI.”
Rose 2015 [45]	Canada	2008–2010	103	95 (48%)	Children	Previous contact	QFT-GIT	15 mm	Not specified	“For close contacts of HS+ individuals, the QFT added little sensitivity to the TST for detection of TB infection. The QFT correlated much better with exposure than the TST, especially in BCG-immunized children, and it has the greatest potential benefit for evaluation of those at lower risk of latent TB infection.”
Seyhan 2010 [46]	Turkey	-	100	67 (67%)	Adults	Previous contact—TB history	QFT-GIT	10 mm	Hemodialysis	“QFT-G test results were more closely associated with TB risk factors than were positive TST results. Additionally, the QFT-G test was not affected by BCG vaccination. We concluded that the QFT-G test is a more useful diagnostic method than for detecting LTBI in HD patients.”
Şimşek 2010 [47]	Turkey	2007–2008	95	81 (85%)	Adults	Previous contact	QFT-GIT-T.SPOT	15 mm	Not specified	“IFN-γ tests could be useful in diagnosing LTBI and chemo-prophylaxis, as the false negativity of the TST was higher compared to both QFT-G and T-SPOT.TB. However, additional studies are needed to assess better the utility of these tests with large populations.”
Song 2014 [48]	South Korea	2008–2012	2982	1818 (61%)	Children	Previous contact	QFT-GIT	15 mm–10 mm	Not specified	“The results of this study suggest that the TST cutoff point for patients aged 11–17 years would be 15 mm in other study. The OR of QFT-GIT for the development of active tuberculosis and its intermediate agreement with TST using a 15 mm cutoff demonstrates its role as an adjunct diagnostic tool to current clinical practice. Positive responders to both TST and QFT-GIT at the outset may benefit from chemoprophylaxis.”
Surve 2021 [49]	India	2019–2021	299	-	Children	Previous contact	QFT-Gold Plus	10 mm	Not specified	“The study strongly recommends both TST and QFT-TB Gold Plus test for the diagnosis of LTBI in under-five children. A moderate concordance in children <24 months endorses the reliability of QFT-TB Gold Plus in diagnosing LTBI in this age group. This study highlights the need for screening undernourished children for LTBI to consider repeating IGRA testing for TST positives as per the window period and risk of ongoing exposure.”
Sutanto 2023 [50]	Indonesia	-	30	-	Adults	Previous contact	T. SPOT	Not specified	Not specified	“There is a substantial level of concordance between TST and T-SPOT.TB examination in detecting LTBI in HCW with a kappa value of 0.603 (0.600 < K < 0.800), $p < 0.001$. The results of close contact diagnostic testing with T-SPOT. TB gets a sensitivity of 60.0% and a specificity value of 86.7%, while the results of the close contact diagnostic test with TST get a sensitivity of 33.3% and a specificity value of 93.3%. TST and T-SPOT. TB diagnostic tools have specificity above 85%, so they are good at determining negative (true negative) results in healthy subjects. There is a correlation between the workplace location (close

										contact) and the T-SPOT.TB examination, the correlation value is moderate (r = 0.436) with $p = 0.008$.”
Triverio 2009 [51]	Switzerland	-	62	14 (23%)	Adults	Previous contact-Chest x-ray suggestive of tuberculosis—Combination	QFT-GIT-T.SPOT	5 mm	Hemodialysis	“In this population, QFT was superior to TST for detecting LTBI, but both IGRAs and TST have important limitations and are unreliable for screening for LTBI.”
Winthrop 2008 [52]	USA	-	100	-	Adults	Previous contact	QFT-GIT-T.SPOT	5 mm	Hemodialysis	“Positive IFN assay results were more closely associated with recent TB exposure than were positive TST results. QFT-G and ELISPOT might offer a better method for detecting TB infection in ESRD patients.”
