# Supplementary figures and images for: Comparative Diagnostic Performance of TST and IGRAs in the Diagnosis of Latent Tuberculosis Infection: A Systematic Review and Diagnostic Meta-Analysis
Source: Diagnostics (Basel). 2026 Mar 23;16(6):951. doi: 10.3390/diagnostics16060951 (PMC13025171; doi:10.3390/diagnostics16060951)

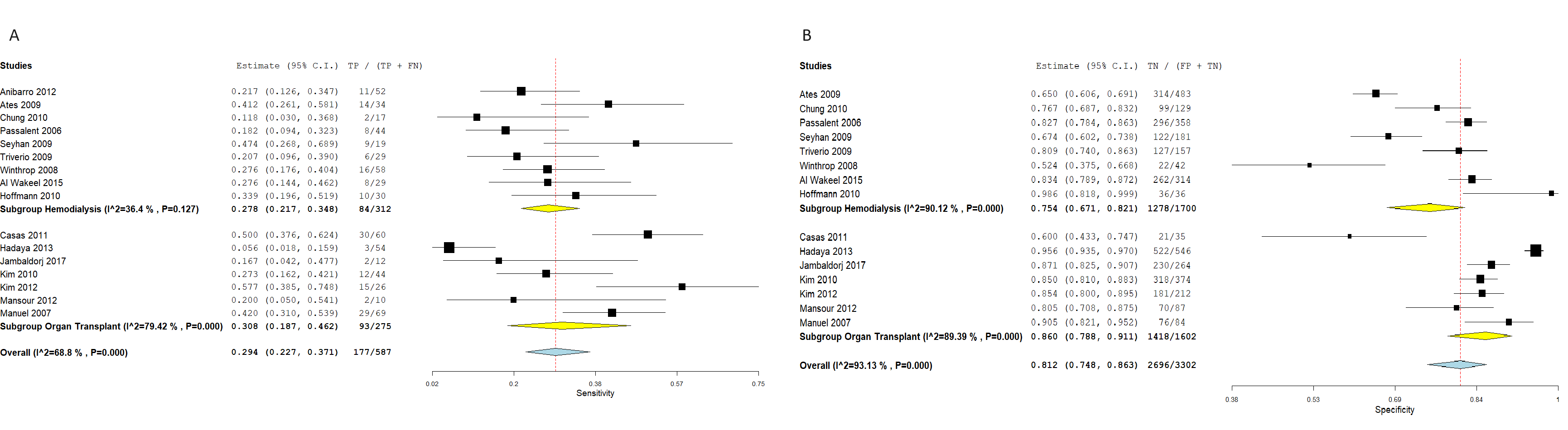

Supplement: Supplementary file 1 [file diagnostics-16-00951-s001.zip › Supplementary Figure S1.png]

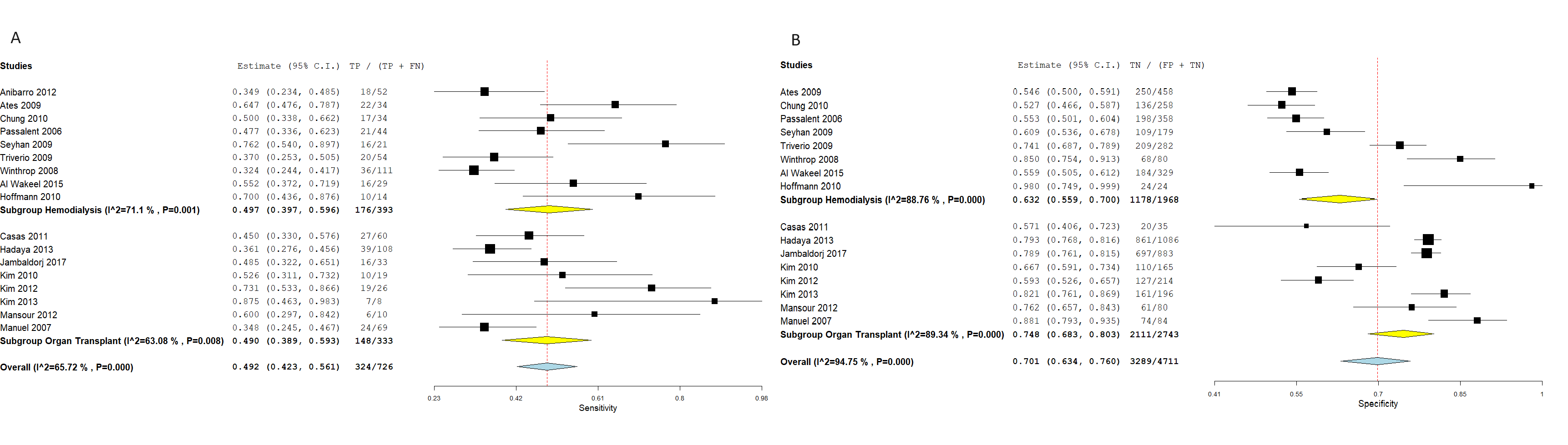

Supplement: Supplementary file 1 [file diagnostics-16-00951-s001.zip › Supplementary Figure S2.png]

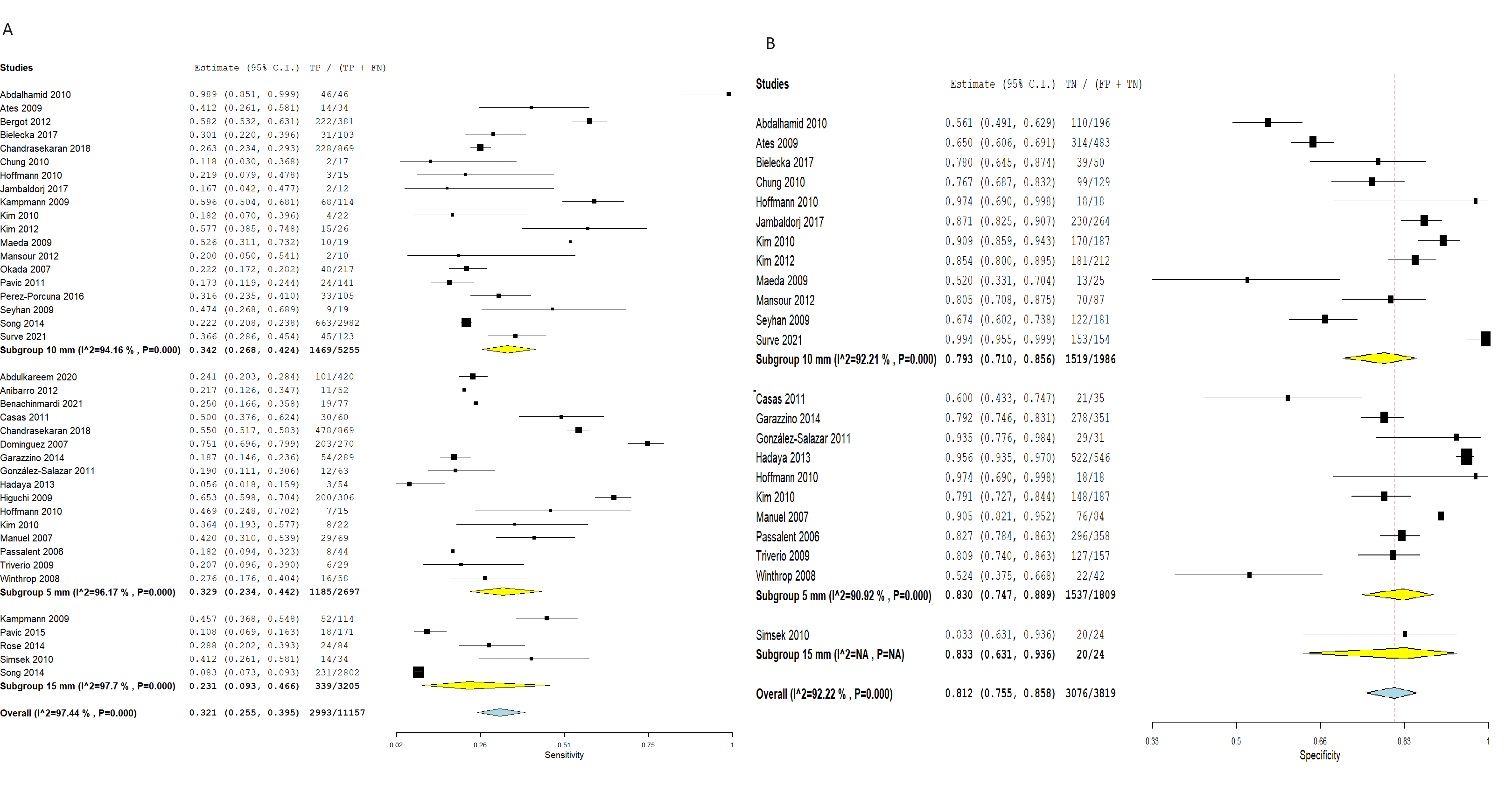

Supplement: Supplementary file 1 [file diagnostics-16-00951-s001.zip › Supplementary Figure S3.png]

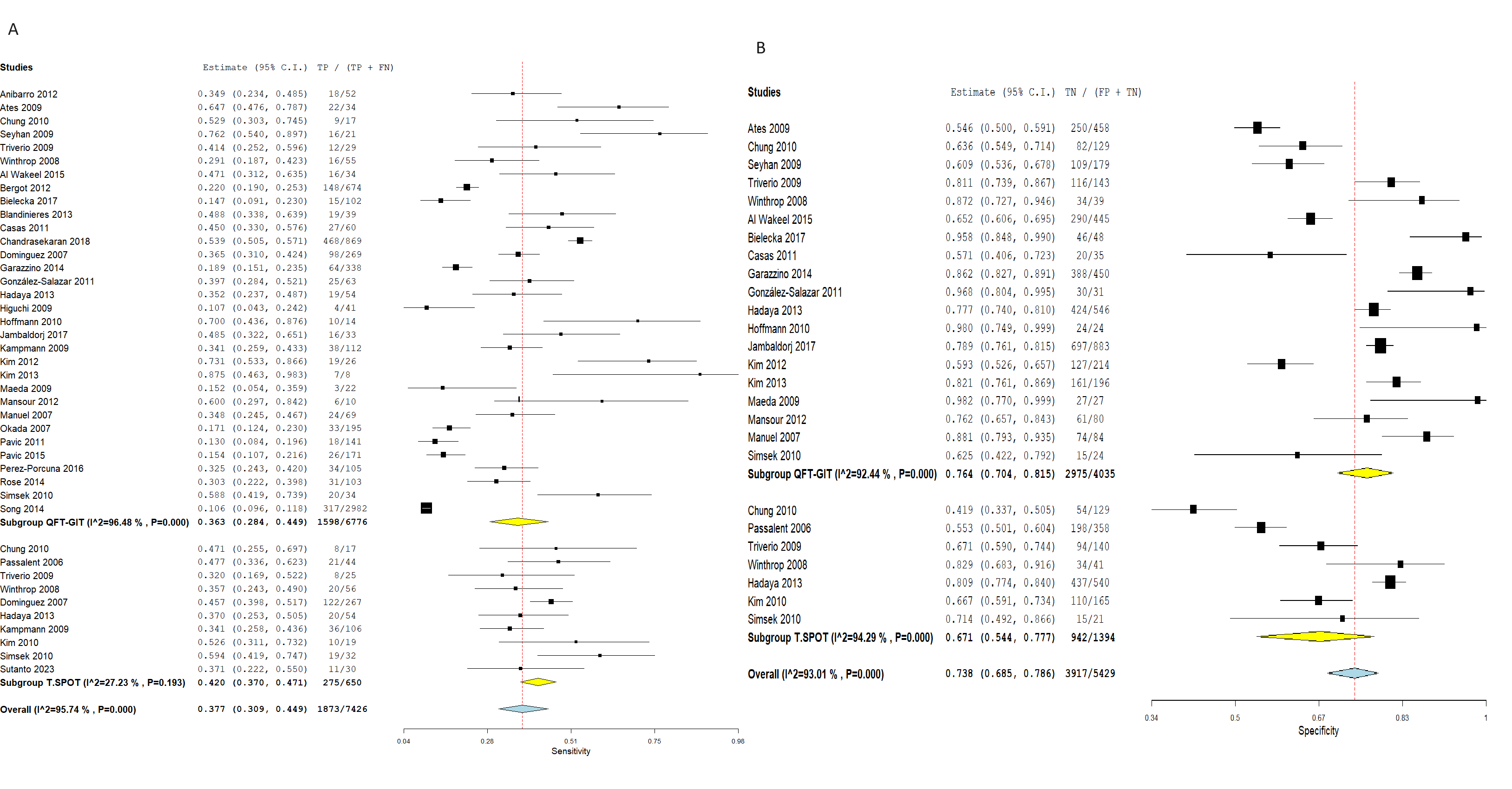

Supplement: Supplementary file 1 [file diagnostics-16-00951-s001.zip › Supplementary Figure S4.png]
